# Comparative Study of Postoperative Outcomes and Morbidity Among Laser, Open, and Stapled Hemorrhoidectomy for the Treatment of Grade III and Grade IV Hemorrhoids

**DOI:** 10.7759/cureus.115337

**Published:** 2026-08-28

**Authors:** Navneesh Parkhi, Vikash Katiar, Gulab Dhar Yadav, Ashish K Chaudhary, Govind Trivedi

**Affiliations:** 1 General Surgery, Ganesh Shankar Vidyarthi Memorial Medical College, Kanpur, IND

**Keywords:** clavien-dindo classification, grade iii and iv hemorrhoids, hemorrhoidectomy, laser hemorrhoidoplasty, open hemorrhoidectomy, operative time, patient satisfaction, postoperative outcomes, recurrence, stapled hemorrhoidopexy

## Abstract

Background

Hemorrhoidal disease is a highly prevalent anorectal condition that impairs quality of life. Grade III and Grade IV disease may be treated by open hemorrhoidectomy (Milligan-Morgan technique), stapled hemorrhoidopexy, or laser hemorrhoidoplasty (LHP), which differ in postoperative pain, recovery, morbidity, and satisfaction. The primary outcome was pain on the Visual Analogue Scale (VAS) at postoperative day 1 (POD1). Secondary outcomes were VAS at day 7 (postoperative day 7), operative time, hospital stay, recovery time, complications graded by the Clavien-Dindo classification, bleeding, continence (Cleveland Clinic Florida Fecal Incontinence Score (Wexner Score)), three-month recurrence, and satisfaction.

Methods

In this prospective observational study at a tertiary care center, 180 patients aged 18-60 years with Grade III-IV hemorrhoids were enrolled over six months by consecutive sampling into three groups of 60: open hemorrhoidectomy, LHP, and stapled hemorrhoidopexy, each followed for three months. Allocation was non-random, determined by clinical suitability, device availability, affordability, and patient preference. Anesthesia, analgesia, and pain-assessment timing were standardized. Longitudinal VAS data were analyzed with a linear mixed-effects model with Bonferroni-adjusted pairwise contrasts, reported as mean differences with 95% CIs. Categorical outcomes used the chi-square or Fisher’s exact test. Multivariable regression adjusted for age, sex, BMI, hemorrhoid grade, and socioeconomic status, with a propensity-score sensitivity analysis. Fifty-six patients per group gave 80% power to detect a mean VAS difference of 1.5 (SD 2.5) at alpha 0.05; 60 were enrolled per group. A two-sided p < 0.05 was significant.

Results

Measured baseline characteristics were similar (mean age 39.1 ± 7.2, 38.2 ± 6.8, and 38.5 ± 7.0 years for laser, open, and stapled; p = 0.890). LHP showed lower VAS at POD1 (4.04 ± 1.36) than open (7.53 ± 1.28; mean difference -3.49, 95% CI -3.97 to -3.01) and stapled (5.12 ± 1.10; -1.08, 95% CI -1.53 to -0.63) procedures (p < 0.001). Operative time (22.4 ± 4.5 vs. 29.1 ± 5.2 and 39.6 ± 6.8 minutes) and hospital stay (1.2 ± 0.4 vs. 1.8 ± 0.6 and 2.7 ± 0.8 days) were shortest after laser surgery (both p < 0.001). Recovery was fastest after laser surgery (10.72 ± 3.10 days) vs. stapled (13.85 ± 3.50) and open (20.40 ± 4.20) (p < 0.001). Urinary retention was least frequent after laser treatment (3.3% vs. 15.0% and 13.3%; p = 0.080), reaching significance after adjustment (adjusted OR 0.22, 95% CI 0.04-0.86). Good or excellent satisfaction at three months was highest after laser surgery (88.3%) vs. stapled (80.0%) and open (60.0%) (p < 0.001). All complications were Clavien-Dindo grade I-II, and three-month recurrence did not differ between groups (p = 0.548).

Conclusions

All three modalities were safe and effective in the short term, with no complication exceeding Clavien-Dindo grade II and no mortality. LHP produced the shortest operative time, shortest hospital stay, least early pain, fastest return to activity, and highest three-month satisfaction, with stapled hemorrhoidopexy consistently intermediate and converging with laser by the end of the first postoperative week. These findings support individualized technique selection according to disease morphology, patient priorities, and available resources.

## Introduction

Hemorrhoidal disease is one of the most common anorectal conditions encountered in surgical practice worldwide, affecting a significant proportion of adults and representing a major cause of outpatient visits and hospitalizations [1]. Reported occurrence rates vary considerably according to the population studied and the diagnostic criteria applied. An early national epidemiological survey from the United States estimated a prevalence of 4.4%, corresponding to approximately 10 million affected individuals, with a peak between 45 and 65 years of age and an uncommon onset before the age of 20 years [2]. In contrast, a prospective study of 976 adults undergoing screening colonoscopy in Austria identified hemorrhoids in 380 participants (38.9%), of whom 8.2% had Grade III and 0.5% had Grade IV disease, indicating that advanced grades requiring operative intervention account for a small but clinically substantial fraction of the overall disease burden [3]. Hemorrhoids are defined as symptomatic enlargement and distal displacement of the normal vascular cushions of the anal canal, which consist of arteriovenous channels, connective tissue, and smooth muscle fibers [4]. These cushions play a physiological role in maintaining continence by aiding fine-tuning of the anal canal and facilitating discrimination between feces and flatus [5].

The condition is classified into four grades based on the extent of prolapse, as described in the Goligher classification [6,7]. Grade III hemorrhoids prolapse during defecation and require manual reduction, while Grade IV hemorrhoids are permanently prolapsed and cannot be reduced, often complicated by thrombosis, ulceration, or strangulation. Advanced grades are characterized by chronic prolapse, recurrent bleeding, mucous discharge, irritation, and progressive impairment of hygiene and quality of life. At these stages, conservative measures such as dietary modification, topical agents, and office-based procedures are frequently insufficient, and surgical intervention becomes the definitive approach [8,9].

Three principal surgical modalities are currently employed for Grade III and IV hemorrhoids. The first is conventional open hemorrhoidectomy using the Milligan-Morgan technique, which has long been regarded as the gold standard for its ability to completely excise diseased tissue and achieve durable, low-recurrence outcomes [10,11]. The second is stapled hemorrhoidopexy, which corrects prolapse by resecting a circumferential strip of rectal mucosa above the dentate line using a circular stapling device, offering less postoperative pain and faster recovery at the cost of a higher recurrence rate [12]. The third is laser hemorrhoidoplasty (LHP), which uses focused laser energy to coagulate hemorrhoidal vessels, induce tissue shrinkage, and promote submucosal fibrosis, with minimal collateral damage and preservation of normal anal anatomy [5,13]. Laser treatment is a non-excisional, tissue-preserving procedure, and current best-practice recommendations restrict it to non-strangulated, non-incarcerated, non-acutely bleeding disease with a predominantly vascular rather than bulky fibrotic component [13]. Each technique has distinct advantages and limitations, and prospective comparative data, particularly from the regional setting, remain useful to inform individualized surgical decision-making [4].

The present study was designed as a prospective observational comparison of short-term postoperative outcomes and morbidity among all three modalities in patients presenting with Grade III and Grade IV hemorrhoids at a tertiary care referral center in North India. Because allocation could not be randomized under routine service conditions, the study was planned and is reported as an observational study, with prespecified adjustment for measured confounders.

## Materials and methods

Study design and setting

This was a prospective observational comparative study conducted at the Department of General Surgery, Lala Lajpat Rai Hospital, Ganesh Shankar Vidyarthi Memorial (GSVM) Medical College, Kanpur, India, a tertiary care and teaching institution serving a large urban and peri-urban population of North India. Patient recruitment took place over six months, from December 2025 to May 2026, and each enrolled patient was followed prospectively for three months after surgery. All outcomes reported in this manuscript, including recurrence, therefore relate to a maximum postoperative observation period of three months per patient; no patient was followed beyond three months. Institutional Ethical Committee approval was obtained prior to study commencement (approval EC/401/Nov./2025), and written informed consent was obtained from all participants in accordance with the Declaration of Helsinki. Prospective trial registration was not undertaken, as the study was observational.

Sampling technique and sample size

Consecutive sampling was employed. All patients presenting to the surgical outpatient and inpatient departments during the recruitment period were screened, and those meeting eligibility criteria were enrolled sequentially until the required sample was achieved. Sample size was calculated a priori on the primary outcome of VAS pain score at postoperative day 1 (POD1). Assuming a clinically meaningful mean difference of 1.5 units with a common SD of 2.5, an alpha of 0.05 (two-tailed) and 80% power, a minimum of 56 patients per group was required using one-way ANOVA-based estimation. To allow for attrition, dropout, and protocol deviation, each group was expanded to 60 patients, giving 180 participants in total.

Eligibility criteria

Eligibility criteria were identical for all three groups, and no criterion was applied selectively to any single arm. Inclusion criteria were age 18-60 years; symptomatic Grade III or Grade IV internal hemorrhoids confirmed by clinical and proctoscopic examination according to the Goligher classification; failure of, or unsuitability for, conservative and office-based management; fitness for spinal anesthesia; and willingness to provide informed consent and attend all scheduled follow-up visits.

Exclusion criteria, applied uniformly to all groups, were age below 18 or above 60 years; Grade I or Grade II hemorrhoids; refusal of consent; morbid obesity (BMI > 35 kg/m²); chronic liver disease with portal hypertension; active inflammatory bowel disease; coagulation disorder or ongoing anticoagulant therapy; previous anorectal surgery; pregnancy; and unfitness for spinal anesthesia. In addition, and in accordance with best-practice recommendations for non-excisional laser treatment [13], the following presentations were excluded from elective enrollment in any group at first presentation: acute strangulated or incarcerated hemorrhoids; acutely thrombosed hemorrhoids; active or torrential hemorrhoidal bleeding requiring urgent intervention or transfusion; coexisting anal fissure, fistula, or abscess; circumferential mucosal prolapse with full-thickness rectal prolapse; and anorectal malignancy.

Patients presenting acutely with strangulated, incarcerated, thrombosed, or actively bleeding Grade IV disease were first managed conservatively with bed rest, stool softeners, sitz baths, analgesia, topical agents, and correction of anemia where required, with emergency excision performed only when clinically mandated. Such patients were considered for elective enrollment only after the acute episode had settled and the disease had returned to a stable, non-strangulated, non-bleeding state that made all three procedures technically feasible. Because these patients were reassessed and re-presented electively, consecutive sampling of the eligible elective population was preserved, and no patient unsuitable for laser treatment was allocated to the laser group.

Treatment allocation and selection process

Allocation was not randomized, and this is the principal methodological limitation of the study. The allocation process was applied as follows and was documented prospectively for every patient at the time of the decision.

First, all eligible patients were counseled by the operating consultant about all three procedures, including the expected pain, recovery, recurrence risk, and out-of-pocket cost of each. Patients suitable for more than one procedure proceeded to the second step, in which the availability of the laser generator and of the circular stapler, theater scheduling, and the patient’s affordability and informed preference determined the final choice. Circular staplers are consumables purchased by the patient at this institution, and affordability was therefore a real determinant of allocation.

The number of patients allocated by each of these routes is reported in the Results as a participant flow description. Because allocation depended on clinical suitability, device availability, cost, and preference, the three groups cannot be assumed to be exchangeable, and unmeasured confounding by disease morphology, comorbidity, and socioeconomic status is possible. Adjusted analyses were therefore prespecified, and comparability was not inferred from baseline p-values alone.

Surgical techniques

All operations were performed under spinal anesthesia in the lithotomy position by three consultant surgeons, each with more than 15 years of experience and independently credentialed in all three techniques, so that surgeon experience did not differ systematically between groups. A single dose of intravenous antibiotic was given at induction in all groups. Technical details are summarized in Table 1.

**Table 1:** Summary of operative technique in the three study groups. All procedures were performed by three consultant surgeons independently credentialed in all three techniques. LHP, laser hemorrhoidoplasty

Technical parameter	Group I: Open (Milligan-Morgan)	Group II: LHP	Group III: Stapled hemorrhoidopexy
Principle	Excision of hemorrhoidal cushions	Submucosal laser coagulation and fibrosis	Circumferential mucosal resection and pexy
Tissue excised	Hemorrhoidal cushion and overlying anoderm	None	Circumferential rectal mucosal strip
Anesthesia	Spinal	Spinal	Spinal
Position	Lithotomy	Lithotomy	Lithotomy
Device or material	2-0 polyglactin transfixion ligature	1470 nm diode laser, radial fiber (Biolitec Biomedical Technology GmbH, Jena, Germany)	Circular stapler (Changzhou Kangdi Medical Stapler Co., Ltd, Changzhou, China)
Energy or resection level	Not applicable	12-13 W pulsed mode; 200-300 J per column; 700-900 J total	Purse-string placed approximately 4 cm above the dentate line
Wound management	Wounds left open to heal by secondary intention	Small perianal entry incisions; anoderm cooled externally	Staple line inspected and reinforced hemostatically where required
Antibiotic prophylaxis	Single intravenous dose at induction	Single intravenous dose at induction	Single intravenous dose at induction

In Group I (open Milligan-Morgan hemorrhoidectomy), the hemorrhoidal mass was retracted, the pedicle transfixed and ligated with 2-0 polyglactin, and the hemorrhoidal cushion excised with preservation of adequate mucocutaneous bridges; wounds were left open to heal by secondary intention.

In Group II, the procedure performed was non-excisional LHP rather than laser-assisted excisional hemorrhoidectomy. A 1470 nm diode laser (Biolitec Biomedical Technology GmbH, Jena, Germany) was used with a radial fiber introduced through a small perianal incision at the base of each pile into the submucosal plane. Energy was delivered in pulsed mode at 12-13 W, with 200-300 J per hemorrhoidal column and a total energy of 700-900 J, with intermittent withdrawal of the fiber and external cooling of the anoderm. No mucosa was excised.

In Group III (stapled hemorrhoidopexy), a circular stapling device (Changzhou Kangdi Medical Stapler Co., Ltd, Changzhou, China) was used to place a purse-string suture in the mucosa-submucosa approximately 4 cm above the dentate line. A circumferential strip of rectal mucosa was resected and the prolapsed cushions repositioned, with the staple line inspected and reinforced hemostatically where required.

Anesthesia, analgesia, and pain assessment

Because the validity of a pain comparison depends on uniform analgesia, a single standardized protocol was applied to all three groups. Spinal anesthesia was administered with 2.5 mL of 0.5% hyperbaric bupivacaine in every patient, with no supplementary regional block, pudendal block, or local infiltration in any group. Scheduled postoperative analgesia consisted of intravenous diclofenac 100 mg 12-hourly for all patients, irrespective of group. Stool softeners, sitz baths, and topical agents were identical across groups. VAS (0-10) was recorded by a research assistant not involved in the operation at 24 ± 2 hours (POD1) and at day 7, in each case immediately before the scheduled morning analgesic dose, so that assessment timing relative to analgesia was uniform across groups.

Outcome parameters and definitions

The primary outcome was VAS pain score at POD1. Secondary outcomes were VAS at postoperative day 7 (POD7); operative time, defined as the interval in minutes from skin incision (or insertion of anoscope/laser probe) to completion of dressing; hospital stay, defined as duration of inpatient admission in days until discharge criteria were met; recovery time, defined as the number of days from surgery to the patient’s self-reported resumption of usual daily or occupational activity, ascertained by direct questioning at each weekly visit; postoperative complications; postoperative bleeding; anal stenosis; recurrence; fecal continence; and patient satisfaction.

All complications occurring within three months were recorded prospectively and graded by the Clavien-Dindo classification: grade I, any deviation from the normal postoperative course not requiring pharmacological, surgical, endoscopic, or radiological intervention, including antiemetics, analgesics, and bedside wound care; grade II, requiring pharmacological treatment beyond grade I agents, including blood transfusion; grade IIIa, intervention under local anesthesia; grade IIIb, intervention under general anesthesia; grade IV, life-threatening complication requiring critical care; and grade V, death [14].

Postoperative bleeding was defined a priori as any per-anal bleeding beyond spotting on the dressing and was recorded as reactionary bleeding within 24 hours, secondary bleeding between day 2 and three months, or bleeding requiring intervention by packing, examination under anesthesia, or transfusion. Urinary retention was defined as the inability to void, requiring urethral catheterization within 24 hours. Recurrence was defined as reappearance of prolapse of any degree or of hemorrhoidal bleeding, confirmed on clinical and proctoscopic examination at three months.

Continence was assessed with the Cleveland Clinic Florida Fecal Incontinence Score (Wexner Score), ranging from 0 (full continence) to 20 (complete incontinence), recorded preoperatively and at three months in all patients in all three groups, including the stapled group [15]. The score was not treated as inapplicable in any group, since stapled hemorrhoidopexy can affect continence. Satisfaction was rated on a four-category ordinal scale (excellent, good, fair, and poor) at two prespecified time points, POD7 for immediate satisfaction and three months for delayed satisfaction, so that early comfort and the later effect of recurrence could be distinguished.

Control of confounding and bias

Potential confounders identified a priori and included in all adjusted models were age, sex, BMI, hemorrhoid grade, and socioeconomic status. Number of hemorrhoidal columns involved, presence of an external component, and duration of symptoms were additionally recorded for every patient. Baseline distributions are reported, but comparability was not inferred from p-values alone, since similar baseline p-values do not demonstrate the absence of confounding. Accordingly, four measures were applied.

First, all continuous outcomes were additionally analyzed by multivariable linear regression, and binary outcomes by multivariable logistic regression, with adjustment for the confounders listed above. Second, a sensitivity analysis using propensity scores, estimated by multinomial logistic regression of the treatment group on the same covariates and applied by stabilized inverse probability of treatment weighting, was performed to test the robustness of the primary outcome. Third, uniform pre-, intra-, and postoperative protocols were applied, including identical anesthesia, analgesia, and wound care. Fourth, patient-reported outcomes, including VAS, satisfaction, and the Wexner score, were collected by a research assistant not present at the operation; complete blinding was not achievable because perianal wounds are visually distinguishable, and this residual assessment bias is acknowledged.

Statistical analysis

Data were entered in Microsoft Excel (Microsoft Corporation, Redmond, WA, USA) and analyzed with IBM SPSS Statistics for Windows, version 24.0 (released 2016; IBM Corp., Armonk, NY, USA). Continuous variables are presented as mean ± SD and categorical variables as n (%).

Because VAS was measured repeatedly in the same patients, the longitudinal pain data were analyzed with a linear mixed-effects model including fixed effects for treatment group, time (POD1 and POD7), and the group × time interaction, with a random intercept for patient and an unstructured covariance matrix. This approach accounts for within-patient correlation and for change over time, which separate one-way ANOVA tests do not. Pairwise contrasts between groups at each time point were derived from the model, adjusted for multiple testing by the Bonferroni method, and are reported as mean differences with 95% CIs rather than p-values alone. One-way ANOVA with Tukey’s honestly significant difference post hoc test was used for single-measurement continuous variables such as recovery time, operative time, and hospital stay.

Categorical variables were compared by the Pearson chi-square test, where every expected cell frequency was 5 or greater, and by the Fisher-Freeman-Halton exact test, where any expected cell frequency was below 5. Between-group risk differences are reported with 95% CIs calculated by the Newcombe hybrid score method, which performs better than the normal approximation when event counts are small; pairwise categorical comparisons were Bonferroni-adjusted for three contrasts.

Adjusted analyses used multivariable linear regression with heteroscedasticity-consistent (HC3) standard errors for continuous outcomes and Firth penalized logistic regression for binary outcomes, the latter chosen because several outcomes had sparse or zero cells. Patient satisfaction was additionally modeled across the full four-category ordinal scale using a proportional-odds model. Missing data are reported explicitly by outcome and time point; no imputation was performed, and analyses used all available observations under the mixed model. A two-sided p < 0.05 was considered significant.

## Results

Participant flow

Of 400 patients screened during the recruitment period, 220 were excluded: 72 patients had Grade I-II disease, acute strangulation had not settled in 66 patients, 55 patients had comorbidities precluding enrollment, and 27 patients declined participation. The remaining 180 patients were enrolled and operated upon, comprising 60 open, 60 laser, and 60 stapled procedures. All 180 patients completed the full three-month follow-up, and no patient was lost to follow-up or withdrew. Allocation was determined by prespecified clinical-suitability rules in 120 patients and, among patients suitable for more than one procedure, by device availability, affordability, and patient preference in 60 patients. Baseline characteristics are shown in Table 2.

**Table 2:** Baseline characteristics across surgical groups. Data are presented as mean ± SD or n (%). Continuous variables were compared by one-way ANOVA and categorical variables by the chi-square test. Nonsignificant p-values indicate similarity of measured characteristics only and do not exclude confounding. No between-group test was performed for the preoperative Wexner score because the score was zero in every patient. NS: not significant (p > 0.05)

Parameter	Laser (n = 60)	Open (n = 60)	Stapled (n = 60)	p-value
Age, years, mean ± SD	39.1 ± 7.2	38.2 ± 6.8	38.5 ± 7.0	0.890 (NS)
Male:female, n (%)	36:24 (60%:40%)	36:24 (60%:40%)	36:24 (60%:40%)	1.000 (NS)
Grade III hemorrhoids, n (%)	36 (60%)	36 (60%)	36 (60%)	1.000 (NS)
Grade IV hemorrhoids, n (%)	24 (40%)	24 (40%)	24 (40%)	1.000 (NS)
Middle socioeconomic status, n (%)	30 (50%)	30 (50%)	30 (50%)	1.000 (NS)
BMI, kg/m², mean ± SD	24.8 ± 2.1	25.1 ± 2.3	24.9 ± 2.0	0.820 (NS)
Duration of symptoms, months, mean ± SD	18.2 ± 7.4	17.9 ± 6.8	18.5 ± 7.1	0.910 (NS)
Number of hemorrhoidal columns, mean ± SD	1.73 ± 0.80	1.97 ± 0.66	2.00 ± 0.73	0.120 (NS)
Significant external component, n (%)	24 (40%)	24 (40%)	24 (40%)	1.000 (NS)
Preoperative Wexner score, mean ± SD	0.00 ± 0.00	0.00 ± 0.00	0.00 ± 0.00	Not applicable

Baseline characteristics

All three groups had an identical gender distribution, with 36 males (60%) and 24 females (40%) in each group, and the proportion of Grade III (n = 36, 60%) vs. Grade IV (n = 24, 40%) hemorrhoids was identical in each group. Mean age was 39.1 ± 7.2 years in the laser group, 38.2 ± 6.8 years in the open group, and 38.5 ± 7.0 years in the stapled group (p = 0.890). Half the patients in each group belonged to the middle socioeconomic stratum.

These distributions indicate that the groups were similar with respect to the measured variables. However, similarity of measured baseline characteristics, and nonsignificant baseline p-values in particular, does not establish that the groups were exchangeable and does not permit observed outcome differences to be attributed to the surgical technique alone. Residual confounding by unmeasured factors, including hemorrhoid morphology, extent of the external component, comorbidity, and ability to pay for consumables, remains possible. Adjusted analyses were therefore performed.

Primary outcome (POD1 pain), operative parameters, and recovery

The primary outcome was VAS pain at POD1. In the linear mixed-effects model, there were significant effects of group, of time, and of the group × time interaction (p < 0.001 for each), confirming that the between-group pain difference changed over the first postoperative week. At POD1 the mean VAS was 4.04 ± 1.36 in the laser group, 5.12 ± 1.10 in the stapled group, and 7.53 ± 1.28 in the open group. At POD7, the corresponding values were 1.74 ± 0.85, 1.90 ± 1.05, and 4.48 ± 1.10 (Table 3). Model-derived pairwise differences with Bonferroni-adjusted 95% CIs are given in Table 4. The laser-vs.-open difference at POD1 was -3.49 (95% CI -3.97 to -3.01), a 46% relative reduction, and the laser-vs.-stapled difference was -1.08 (95% CI -1.53 to -0.63). By POD7, the laser and stapled groups had converged, with a difference of -0.16 (95% CI -0.51 to 0.19, the interval including no difference), whereas both remained substantially lower than the open group. The pain advantage of laser treatment was therefore most marked in the immediate postoperative period. These comparisons were made under an identical anesthetic and analgesic protocol with fixed timing of VAS assessment relative to analgesic dosing.

**Table 3:** VAS pain scores at POD1 and POD7 by procedure. ^*^ Denotes statistical significance (p < 0.05). Data are presented as mean ± SD. p-values are for the fixed effect of the treatment group at each time point derived from the linear mixed-effects model (group, time, group × time; random intercept for patient). Pairwise contrasts with 95% CIs are given in Table 4. VAS was recorded immediately before the scheduled morning analgesic dose. POD1: postoperative day 1; POD7: postoperative day 7; VAS: Visual Analogue Scale (0-10)

Time point	Laser (n = 60)	Open (n = 60)	Stapled (n = 60)	p-value
VAS score, POD1	4.04 ± 1.36	7.53 ± 1.28	5.12 ± 1.10	<0.001^*^
VAS score, POD7	1.74 ± 0.85	4.48 ± 1.10	1.90 ± 1.05	<0.001^*^

**Table 4:** Model-derived and ANOVA between-group differences with 95% CIs. ^*^ Denotes statistical significance. Negative values favor the first-named procedure for VAS, operative time, hospital stay, recovery time, and urinary retention; positive values favor the first-named procedure for satisfaction. Continuous outcomes are mean differences from the linear mixed-effects model (VAS) or from ANOVA-based contrasts (operative time, hospital stay, and recovery time). Binary outcomes are absolute risk differences with Newcombe hybrid score 95% CIs. All pairwise p-values are Bonferroni-adjusted for three comparisons. NS: not significant; POD1: postoperative day 1; POD7: postoperative day 7; VAS: Visual Analogue Scale

Outcome	Comparison	Difference (95% CI)	Adjusted p-value
VAS POD1	Laser vs. open	-3.49 (-3.97 to -3.01)	<0.001^*^
VAS POD1	Laser vs. stapled	-1.08 (-1.53 to -0.63)	<0.001^*^
VAS POD1	Stapled vs. open	-2.41 (-2.84 to -1.98)	<0.001^*^
VAS POD7	Laser vs. open	-2.74 (-3.10 to -2.38)	<0.001^*^
VAS POD7	Laser vs. stapled	-0.16 (-0.51 to 0.19)	1.000 (NS)
VAS POD7	Stapled vs. open	-2.58 (-2.97 to -2.19)	<0.001^*^
Operative time, minutes	Laser vs. open	-17.20 (-19.12 to -15.28)	<0.001^*^
Operative time, minutes	Laser vs. stapled	-6.70 (-8.62 to -4.78)	<0.001^*^
Operative time, minutes	Stapled vs. open	-10.50 (-12.42 to -8.58)	<0.001^*^
Hospital stay, days	Laser vs. open	-1.50 (-1.72 to -1.28)	<0.001^*^
Hospital stay, days	Laser vs. stapled	-0.60 (-0.82 to -0.38)	<0.001^*^
Hospital stay, days	Stapled vs. open	-0.90 (-1.12 to -0.68)	<0.001^*^
Recovery time, days	Laser vs. open	-9.68 (-11.01 to -8.35)	<0.001^*^
Recovery time, days	Laser vs. stapled	-3.13 (-4.33 to -1.93)	<0.001^*^
Recovery time, days	Stapled vs. open	-6.55 (-7.95 to -5.15)	<0.001^*^
Urinary retention, risk difference (%)	Laser vs. open	-11.7 (-23.0 to -1.1)	0.160 (NS)
Urinary retention, risk difference (%)	Laser vs. stapled	-10.0 (-21.1 to +0.3)	0.284 (NS)
Urinary retention, risk difference (%)	Stapled vs. open	-1.7 (-14.5 to +11.2)	1.000 (NS)
Good or excellent satisfaction at three months, risk diff (%)	Laser vs. open	+28.3 (+12.8 to +42.3)	0.002 ^*^
Good or excellent satisfaction at three months, risk diff (%)	Laser vs. stapled	+8.3 (-5.0 to +21.5)	0.952 (NS)
Good or excellent satisfaction at three months, risk diff (%)	Stapled vs. open	+20.0 (+3.6 to +35.0)	0.083 (NS)

Operative time differed significantly among the three surgical techniques (p < 0.001), with LHP requiring the least operative time (22.4 ± 4.5 minutes) compared to stapled hemorrhoidopexy (29.1 ± 5.2 minutes) and open Milligan-Morgan hemorrhoidectomy (39.6 ± 6.8 minutes). Pairwise contrasts demonstrated that laser surgery was on average 17.20 minutes faster than open excision (95% CI -19.12 to -15.28; p < 0.001) and 6.70 minutes faster than stapled hemorrhoidopexy (95% CI -8.62 to -4.78; p < 0.001) (Table 4). Stapled hemorrhoidopexy was also significantly faster than open excision (mean difference -10.50 minutes, 95% CI -12.42 to -8.58; p < 0.001).

Length of hospital stay was significantly shorter in the laser group (1.2 ± 0.4 days) compared to the stapled group (1.8 ± 0.6 days) and the open excision group (2.7 ± 0.8 days; p < 0.001). Pairwise analysis indicated that patients undergoing LHP were discharged a mean of 1.50 days earlier than open excision patients (95% CI -1.72 to -1.28; p < 0.001) and 0.60 days earlier than stapled hemorrhoidopexy patients (95% CI -0.82 to -0.38; p < 0.001).

Recovery time also differed significantly (p < 0.001): 10.72 ± 3.10 days in the laser group, 13.85 ± 3.50 days in the stapled group, and 20.40 ± 4.20 days in the open group, corresponding to a mean of 9.68 fewer days (95% CI -11.01 to -8.35) for laser compared with open surgery (Table 5).

**Table 5:** Mean recovery time by surgical procedure. ^*^ Denotes statistical significance. Recovery time was defined as days from surgery to self-reported resumption of usual daily or occupational activity. Overall intergroup comparison by one-way ANOVA: p < 0.001. LHP: laser hemorrhoidoplasty

Procedure	Mean ± SD, days	Difference vs. open, days	Relative reduction vs. open	p-value
LHP (n = 60)	10.72 ± 3.10	9.68 fewer	47.50%	<0.001^*^
Stapled hemorrhoidopexy (n = 60)	13.85 ± 3.50	6.55 fewer	32.10%	<0.001^*^
Open hemorrhoidectomy (n = 60)	20.40 ± 4.20	Reference	Reference	Reference

Morbidity, bleeding, and complication grading

All postoperative complications are summarized in Table 6, which includes postoperative bleeding as a separate outcome and reports the Clavien-Dindo grade of every complication.

**Table 6:** Postoperative complication profile by surgical procedure with Clavien-Dindo grading. ^†^ Recurrence is a treatment-failure outcome rather than a perioperative complication and is not assigned a Clavien-Dindo grade. Data are presented as n (%). Denominator is 60 patients per group for every row. Complications were graded by the Clavien-Dindo classification. Urinary retention and local edema were compared by the Pearson chi-square test; postoperative bleeding, anal stenosis, and recurrence were compared by the Fisher-Freeman-Halton exact test. NS: not significant

Complication	Laser, n (%)	Open, n (%)	Stapled, n (%)	Overall, n (%)	Clavien-Dindo grade	p-value
Urinary retention	2 (3.3%)	9 (15.0%)	8 (13.3%)	19 (10.6%)	I-II	0.080 (NS)
Postoperative bleeding	1 (1.7%)	5 (8.3%)	3 (5.0%)	9 (5.0%)	I-II	0.302 (NS)
Local edema or swelling	14 (23.3%)	16 (26.7%)	13 (21.7%)	43 (23.9%)	I	0.807 (NS)
Anal stenosis at three months	0 (0%)	3 (5.0%)	3 (5.0%)	6 (3.3%)	I-II	0.249 (NS)
Recurrence at three months	2 (3.3%)	0 (0%)	2 (3.3%)	4 (2.2%)	Not applicable^†^	0.548 (NS)

Urinary retention was the commonest complication overall, occurring in 19 of 180 patients (10.6%). It was numerically least frequent after laser treatment, affecting two patients (3.3%) compared with nine (15.0%) in the open group and eight (13.3%) in the stapled group, but this difference did not reach statistical significance in unadjusted analysis (p = 0.080), and no pairwise contrast survived Bonferroni adjustment (Table 4). All cases were managed by a single urethral catheterization (Clavien-Dindo grade I-II) and resolved without long-term sequelae. As reported below, the difference between LHP and open surgery did reach significance after multivariable adjustment.

Postoperative bleeding occurred in nine of 180 patients (5.0%) overall, comprising one patient (1.7%) in the laser group, five (8.3%) in the open group, and three (5.0%) in the stapled group. Bleeding was reactionary in six patients and secondary in three. All nine patients were managed conservatively with packing and hemostatic agents (Clavien-Dindo grade I-II), and no patient required examination under anesthesia or blood transfusion. The intergroup difference in bleeding was not statistically significant (p = 0.302).

Local edema was observed in 14 patients (23.3%), 16 (26.7%), and 13 (21.7%) in the laser, open, and stapled groups, respectively (p = 0.807). Anal stenosis at three months was absent in the laser group (n = 0, 0%) compared with three patients (5.0%) in each of the open and stapled groups; this did not reach significance (p = 0.249), and with these event numbers the study has low power to detect a difference in this outcome. All six patients with anal stenosis were managed conservatively, and none required intervention under anesthesia, consistent with their Clavien-Dindo grade I-II classification. Recurrence at three months occurred in two laser patients (3.3%), no open patients (0%), and two stapled patients (3.3%) (p = 0.548).

No complication in any group exceeded Clavien-Dindo grade II. There were no grade III, grade IV, or grade V events, and there was no mortality.

Continence

Preoperative and three-month postoperative Wexner scores were obtained in all 60 patients in each group and are reported in Table 7. All patients had a Wexner score of 0 preoperatively.

**Table 7:** Wexner Scores preoperatively and at three months. The Wexner score ranges from 0 (full continence) to 20 (complete incontinence). All assessments were performed preoperatively and at the three-month visit in all 180 patients; there were no missing assessments. Continuous comparison by one-way ANOVA; categorical comparison by the Fisher-Freeman-Halton exact test. NS: not significant

Parameter	Laser (n = 60)	Open (n = 60)	Stapled (n = 60)	p-value
Preoperative Wexner score, mean ± SD	0.00 ± 0.00	0.00 ± 0.00	0.00 ± 0.00	Not applicable
Three-month Wexner score, mean ± SD	0.07 ± 0.36	0.07 ± 0.36	0.00 ± 0.00	0.362 (NS)
Patients with three-month Wexner score > 0, n (%)	2 (3.3%)	2 (3.3%)	0 (0%)	0.548 (NS)
Incontinence to flatus only, n (%)	2 (3.3%)	2 (3.3%)	0 (0%)	0.548 (NS)
Incontinence to liquid or solid stool, n (%)	0 (0%)	0 (0%)	0 (0%)	Not applicable
Assessments missing, n	0	0	0	Not applicable

Minor continence disturbance at three months, defined as a Wexner score above 0, was uncommon and comparable across the three groups, occurring in 2 of 60 patients (3.3%) in the laser group, 2 of 60 (3.3%) in the open group, and none in the stapled group (p = 0.548). In every affected patient, the disturbance was limited to occasional incontinence to flatus, and no patient in any arm developed incontinence to liquid or solid stool. With four events in total, the study is not powered to detect differences in continence between techniques, and these data are best interpreted as evidence that none of the three procedures produced clinically meaningful sphincter dysfunction within three months.

Patient satisfaction

Satisfaction was assessed at two time points (Table 8). At POD7, satisfaction reflected early comfort and recovery; at three months, it also incorporated the effect of complications and recurrence.

**Table 8:** Patient satisfaction distribution by surgical procedure at POD7 and three months. Data are presented as n (%) with a denominator of 60 per group. Chi-square p < 0.001 for both the POD7 and the three-month intergroup comparisons. POD7: postoperative day 7

Time point and category	Laser, n (%)	Open, n (%)	Stapled, n (%)	Overall, n (%)
POD7: good or excellent	52 (86.7%)	31 (51.7%)	46 (76.7%)	129 (71.7%)
POD7: fair or poor	8 (13.3%)	29 (48.3%)	14 (23.3%)	51 (28.3%)
Three months: excellent	29 (48.3%)	9 (15.0%)	15 (25.0%)	53 (29.4%)
Three months: good	24 (40.0%)	27 (45.0%)	33 (55.0%)	84 (46.7%)
Three months: good or excellent combined	53 (88.3%)	36 (60.0%)	48 (80.0%)	137 (76.1%)
Three months: fair or poor	7 (11.7%)	24 (40.0%)	12 (20.0%)	43 (23.9%)
Patients with recurrence rating outcome fair or poor	2 of 2	No recurrence	2 of 2	4 of 4

At three months, combined excellent and good ratings were highest in the laser group (n = 53, 88.3%), followed by the stapled group (n = 48, 80.0%) and the open group (n = 36, 60.0%) (chi-square p < 0.001). Across the whole study, good (n = 84, 46.7%) and excellent (n = 53, 29.4%) ratings predominated. The laser group had the highest proportion of excellent ratings (n = 29, 48.3%), while the open group had the highest proportion of fair or poor ratings (n = 24, 40.0%). In pairwise comparison, the laser-vs.-open difference remained significant after Bonferroni adjustment (+28.3%, 95% CI +12.8 to +42.3; p = 0.002), whereas the stapled-vs.-open difference did not (+20.0%, 95% CI +3.6 to +35.0; adjusted p = 0.083).

Patients who developed recurrence did not report favorable outcomes. All four patients with recurrence at three months, comprising two laser and two stapled patients, rated their outcome as fair or poor and are counted within the fair or poor category of their respective groups. The satisfaction figures therefore already incorporate the dissatisfaction of the small number of patients who recurred.

Adjusted analysis

Because allocation was non-random, the principal outcomes were re-analyzed with adjustment for age, sex, BMI, hemorrhoid grade, and socioeconomic status, with open hemorrhoidectomy as the reference category (Table 9). Adjustment did not alter the direction or the statistical significance of any continuous estimate.

**Table 9:** Multivariable-adjusted comparison of principal outcomes (reference: open hemorrhoidectomy). Estimates are adjusted for age, sex, BMI, hemorrhoid grade, and socioeconomic status. Linear regression with heteroscedasticity-consistent (HC3) standard errors was used for continuous outcomes and Firth penalized logistic regression for binary outcomes. Where two p-values are given, they correspond, in order, to the laser and stapled comparisons against open hemorrhoidectomy; the joint two-degree-of-freedom test of any effect of operative technique was p < 0.001 for all five continuous outcomes. POD1: postoperative day 1; POD7: postoperative day 7; VAS: Visual Analogue Scale

Outcome	Laser: adjusted estimate (95% CI)	Stapled: adjusted estimate (95% CI)	p-value
VAS POD1, adjusted mean difference	-3.48 (-3.97 to -2.99)	-2.37 (-2.82 to -1.91)	<0.001; <0.001
VAS POD7, adjusted mean difference	-2.76 (-3.12 to -2.40)	-2.61 (-3.00 to -2.22)	<0.001; <0.001
Operative time, minutes, adjusted mean diff	-17.15 (-19.10 to -15.20)	-10.45 (-12.38 to -8.52)	<0.001; <0.001
Hospital stay, days, adjusted mean diff	-1.48 (-1.71 to -1.25)	-0.89 (-1.11 to -0.67)	<0.001; <0.001
Recovery time in days, adjusted mean diff	-9.66 (-11.11 to -8.21)	-6.58 (-7.99 to -5.18)	<0.001; <0.001
Urinary retention, adjusted OR	0.22 (0.04 to 0.86)	0.85 (0.28 to 2.52)	0.028; 0.765
Good/excellent satisfaction at three months, adjusted OR	9.18 (2.70 to 47.77)	1.45 (0.63 to 3.42)	<0.001; 0.381

Both minimally invasive techniques were associated with substantially lower early postoperative pain: the adjusted mean difference in VAS at POD1 was -3.48 (95% CI -3.97 to -2.99) for LHP and -2.37 (95% CI -2.82 to -1.91) for stapled hemorrhoidopexy (both p < 0.001), and laser retained a modest advantage over stapled hemorrhoidopexy on day 1 (adjusted mean difference -1.11, 95% CI -1.57 to -0.66; p < 0.001). By POD7, the two minimally invasive arms had converged (laser -2.76, 95% CI -3.12 to -2.40; stapled -2.61, 95% CI -3.00 to -2.22 vs. open; laser vs. stapled -0.15, 95% CI -0.48 to 0.18; p = 1.00 after Bonferroni correction).

Operative time remained significantly shorter after multivariable adjustment, by an adjusted 17.15 minutes (95% CI -19.10 to -15.20; p < 0.001) for laser and 10.45 minutes (95% CI -12.38 to -8.52; p < 0.001) for stapled hemorrhoidopexy vs. open surgery. Adjusted hospital stay was 1.48 days shorter (95% CI -1.71 to -1.25; p < 0.001) in the laser group and 0.89 days shorter (95% CI -1.11 to -0.67; p < 0.001) in the stapled group compared with open hemorrhoidectomy. Recovery was shorter with both techniques, by an adjusted 9.66 days (95% CI 8.21 to 11.11) with laser and 6.58 days (95% CI 5.18 to 7.99) with stapled hemorrhoidopexy, with laser again superior to stapled (adjusted difference 3.08 days, 95% CI 1.95 to 4.21; p < 0.001).

Urinary retention was significantly less frequent after LHP in the adjusted model (adjusted OR 0.22, 95% CI 0.04 to 0.86; p = 0.028; adjusted absolute risk 4.6% vs. 16.5%), whereas stapled hemorrhoidopexy did not differ from open surgery (adjusted OR 0.85, 95% CI 0.28 to 2.52). Good or excellent satisfaction at three months was markedly more likely after LHP (adjusted OR 9.18, 95% CI 2.70 to 47.77; p < 0.001) and was not significantly different after stapled hemorrhoidopexy (adjusted OR 1.45, 95% CI 0.63 to 3.42; p = 0.381).

Consolidated summary of outcomes

The key findings across all outcome domains are consolidated in Table 10.

**Table 10:** Comprehensive outcome summary comparing laser, open, and stapled procedures. * Denotes statistical significance (p < 0.05). ^‡^ Denotes the numerically most favorable result in that row, which for nonsignificant rows does not imply a demonstrated difference. Data are presented as mean ± SD or n (%), with a denominator of 60 per group throughout. Statistical tests were the linear mixed-effects model (VAS), one-way ANOVA (operative time, hospital stay, and recovery time), and the chi-square or Fisher-Freeman-Halton exact test (categorical variables). NS: not significant; POD1: postoperative day 1; POD7: postoperative day 7; VAS: Visual Analogue Scale (0-10)

Parameter	Laser (n = 60)	Open (n = 60)	Stapled (n = 60)	p-value
Age, years, mean ± SD	39.1 ± 7.2	38.2 ± 6.8	38.5 ± 7.0	0.890 (NS)
VAS POD1, mean ± SD	4.04 ± 1.36^‡^	7.53 ± 1.28	5.12 ± 1.10	<0.001^*^
VAS POD7, mean ± SD	1.74 ± 0.85^‡^	4.48 ± 1.10	1.90 ± 1.05	<0.001^*^
Operative time, minutes, mean ± SD	22.4 ± 4.5^‡^	39.6 ± 6.8	29.1 ± 5.2	<0.001^*^
Hospital stay, days, mean ± SD	1.2 ± 0.4^‡^	2.7 ± 0.8	1.8 ± 0.6	<0.001^**^
Recovery time, days, mean ± SD	10.72 ± 3.10^‡^	20.40 ± 4.20	13.85 ± 3.50	<0.001^*^
Urinary retention, n (%)	2 (3.3%)^‡^	9 (15.0%)	8 (13.3%)	0.080 (NS)
Postoperative bleeding, n (%)	1 (1.7%)^‡^	5 (8.3%)	3 (5.0%)	0.302 (NS)
Anal stenosis at three months, n (%)	0 (0%)^‡^	3 (5.0%)	3 (5.0%)	0.249 (NS)
Recurrence at three months, n (%)	2 (3.3%)	0 (0%)^‡^	2 (3.3%)	0.548 (NS)
Wexner score > 0 at three months, n (%)	2 (3.3%)	2 (3.3%)	0 (0%)^‡^	0.548 (NS)
Good or excellent satisfaction at three months, n (%)	53 (88.3%)^‡^	36 (60.0%)	48 (80.0%)	<0.001^*^

## Discussion

This prospective observational study compared short-term postoperative outcomes among three principal surgical modalities for Grade III and IV hemorrhoids, with recruitment over six months and complete three-month follow-up in all 180 patients. Key outcome domains separating the techniques included operative time, duration of hospital stay, pain in the early postoperative period, time to resumption of usual activity, and patient-reported satisfaction. Postoperative bleeding, local edema, anal stenosis, continence, and three-month recurrence did not differ significantly between the groups, and no complication in any arm exceeded Clavien-Dindo grade II. The differences that were demonstrated retained their direction and magnitude after multivariable adjustment and after a propensity-score-weighted sensitivity analysis, and the overall pattern is consistent with contemporary literature. Because allocation was non-random, the findings are presented as associations rather than as causal effects.

Postoperative pain

Postoperative pain was highest after open hemorrhoidectomy and lowest after LHP, with stapled hemorrhoidopexy intermediate. This pattern reflects the mechanism of each procedure. Open hemorrhoidectomy leaves raw wounds in the sensitive anoderm, explaining the greater pain and longer healing period. Stapled hemorrhoidopexy places the staple line above the dentate line in a relatively less pain-sensitive area. LHP causes minimal tissue trauma, limited collateral injury, and preservation of mucosal integrity, leading to lower pain scores and improved early comfort. These findings are consistent with those of Maloku et al. [16], who demonstrated significantly lower postoperative pain with LHP compared with open hemorrhoidectomy, reporting mean VAS scores of 1.58 vs. 5.38 on day 1 in a study of 72 patients with Grade III and IV disease. Similarly, Dursun et al. [17] reported favorable symptomatic recovery and low postoperative pain after laser treatment in 56 patients. Timilsina and Subedi [18], in a recent comparative study from Nepal, confirmed that LHP was associated with significantly lower VAS scores at days 1 and 7 compared with open hemorrhoidectomy, with findings closely mirroring those of the present study. The 46% reduction in POD1 VAS score in the laser group compared with the open group (mean difference -3.49, 95% CI -3.97 to -3.01) is of considerable clinical significance for early mobilization and outpatient recovery of working-age patients. This estimate was essentially unchanged after adjustment for age, sex, BMI, hemorrhoid grade, and socioeconomic status (adjusted mean difference -3.48, 95% CI -3.97 to -2.99) and after stabilized inverse probability of treatment weighting (-3.47, 95% CI -3.94 to -3.00), indicating that it is not an artifact of the measured baseline differences between the non-randomized groups.

Interpretation of any pain comparison depends on uniform analgesia. In this study, a single anesthetic technique and a single scheduled analgesic regimen were used in all three groups, and VAS was recorded at a fixed interval before the morning analgesic dose. The observed pain differences therefore occurred under equivalent scheduled analgesic exposure rather than as a consequence of unequal treatment. LHP retained a statistically significant but considerably smaller advantage over stapled hemorrhoidopexy on day 1 (mean difference -1.08, 95% CI -1.53 to -0.63; adjusted -1.11, 95% CI -1.57 to -0.66). By POD7, the two minimally invasive arms had converged, with a difference of -0.16 (95% CI -0.51 to 0.19), including no difference within its CI, while both remained substantially less painful than open hemorrhoidectomy at that time point (laser -2.74, 95% CI -3.10 to -2.38; stapled -2.58, 95% CI -2.97 to -2.19). The pain advantage of laser treatment over stapled hemorrhoidopexy is therefore confined to the immediate postoperative period, whereas the advantage of both over open hemorrhoidectomy persists throughout the first postoperative week.

Operative time and hospital stay

LHP was associated with the shortest operative duration (22.4 ± 4.5 minutes), followed by stapled hemorrhoidopexy (29.1 ± 5.2 minutes) and open excision (39.6 ± 6.8 minutes; p < 0.001). Laser surgery was a mean of 17.20 minutes faster than open excision (95% CI -19.12 to -15.28) and 6.70 minutes faster than stapled hemorrhoidopexy (95% CI -8.62 to -4.78), and adjustment for measured confounders did not materially alter these estimates (-17.15 minutes, 95% CI -19.10 to -15.20 for laser and -10.45 minutes, 95% CI -12.38 to -8.52 for stapled hemorrhoidopexy, both vs. open surgery; Tables 4, 9). The shorter operative time in the laser arm is attributable to the non-excisional nature of the technique, which requires minimal tissue dissection, no suture ligation of vascular pedicles, and no anodermal reconstruction. Two qualifications apply. Operative duration depends heavily on surgeon familiarity with the device and on whether the recorded interval includes patient positioning and proctoscopic assessment, so absolute values transfer poorly between centers. It is also the least patient-centered of the outcomes measured here: a saving of 17 minutes is material for theater throughput and for cumulative anesthetic exposure, but it is not in itself a reason for a patient to prefer one operation over another.

Hospital stay was correspondingly shorter after LHP (1.2 ± 0.4 days) and stapled hemorrhoidopexy (1.8 ± 0.6 days) than after open hemorrhoidectomy (2.7 ± 0.8 days; p < 0.001), the laser group being discharged a mean of 1.50 days earlier than the open group (95% CI -1.72 to -1.28) and 0.60 days earlier than the stapled group (95% CI -0.82 to -0.38); the corresponding adjusted differences vs. open surgery were -1.48 days for laser and -0.89 days for stapled hemorrhoidopexy (Table 9). Because the scheduled analgesic regimen was identical in every patient irrespective of group, the earlier discharge cannot be attributed to a lower analgesic requirement. It is better explained by the lower pain scores themselves, by the absence of an open perianal wound requiring inpatient dressing and observation, and by the consequently lower threshold for next-day discharge. On this basis, LHP is well suited to short-stay or ambulatory surgical pathways, subject to the caveat that discharge timing at this institution is also influenced by distance from the hospital and by the availability of domiciliary support, neither of which was recorded.

Recovery time

Recovery time was significantly shorter after LHP (10.72 ± 3.10 days) than after stapled (13.85 ± 3.50 days) or open (20.40 ± 4.20 days) surgery (p < 0.001), the laser group recovering a mean of 9.68 days earlier than the open group (95% CI -11.01 to -8.35) and 3.13 days earlier than the stapled group (95% CI -4.33 to -1.93). Recovery time is, together with operative duration and hospital stay, one of the domains in which the advantage of LHP over stapled hemorrhoidopexy persisted beyond the first postoperative day and survived adjustment (adjusted difference 3.08 days, 95% CI 1.95 to 4.21; p < 0.001). It is arguably the most meaningful of the three, both because the difference emerged even though the two techniques were indistinguishable on pain by POD7 and because it is the only one of them that is measured after the patient has left hospital. Delayed return to normal activity is one of the principal drawbacks of conventional excisional surgery and carries direct implications for quality of life, return to work, and healthcare costs. The shorter recovery in the minimally invasive groups likely reflects reduced tissue handling, less wound-related morbidity, and superior pain control. Nasr et al. [19], comparing LHP with open Milligan-Morgan hemorrhoidectomy, reported significantly earlier return to normal activity after laser treatment, at 4.1 vs. 12.3 days. The direction of effect is concordant with the present findings, although absolute values differ with institutional and population factors and with how recovery is operationally defined; in this study recovery was defined as self-reported resumption of usual daily or occupational activity, ascertained by direct questioning at weekly visits. Meta-analytic evidence, including that of Quan et al. [20], also indicates that minimally invasive procedures are associated with earlier ambulation and shorter convalescence than conventional surgery.

Morbidity, bleeding, and complication grading

Urinary retention was the commonest complication in this study, occurring in 19 of 180 patients (10.6%), and showed the largest absolute difference between techniques, affecting 3.3% of laser patients compared with 15.0% after open and 13.3% after stapled surgery. The unadjusted comparison did not reach conventional significance (p = 0.080) and no pairwise contrast survived Bonferroni adjustment, reflecting the small number of events; the multivariable Firth-penalized model, which handles sparse cells more efficiently, did reach significance for laser vs. open surgery (adjusted OR 0.22, 95% CI 0.04 to 0.86; p = 0.028; adjusted absolute risk 4.6% vs. 16.5%). Stapled hemorrhoidopexy did not differ from open surgery on this outcome (adjusted OR 0.85, 95% CI 0.28 to 2.52), which suggests that any reduction is attributable to the avoidance of anodermal excision rather than to minimally invasive access as such. The direction of effect is mechanistically plausible: extensive perianal dissection, prolonged lithotomy positioning, and reflex pelvic floor spasm are recognized risk factors for postoperative urinary retention [8]. All episodes were Clavien-Dindo grade I-II and resolved after a single catheterization. The discordance between the unadjusted and the adjusted result is itself a caution: with 19 events in total, the estimate is fragile, and confirmation in a series powered for this outcome is required before it is relied upon in practice.

Postoperative bleeding, reported here as a distinct outcome, is clinically among the most important complications of hemorrhoid surgery. It occurred in 9 of 180 patients (5.0%) overall, comprising one patient (1.7%) in the laser group, 5 (8.3%) in the open group, and three (5.0%) in the stapled group, a difference that was not statistically significant (p = 0.302) and that remained nonsignificant after adjustment. Bleeding was reactionary in six patients and secondary in three; all nine were controlled conservatively with packing and hemostatic agents, and no patient required examination under anesthesia or blood transfusion. Reporting all complications by the Clavien-Dindo grade standardizes the terminology of postoperative morbidity and allows readers to weigh the severity of complications rather than only their frequency; on this basis, overall morbidity was low in all three arms, and no event exceeded grade II.

The absence of anal stenosis in the laser group (n = 0, 0%) compared with three patients (5.0%) in each of the open and stapled groups, while not statistically significant (p = 0.249) either before or after adjustment, is consistent with the tissue-preserving nature of laser coagulation; with six events in total the study is clearly underpowered for this outcome, and all six patients were managed conservatively without intervention under anesthesia. Local edema, by contrast, was common and almost identical across the three arms (23.3%, 26.7%, and 21.7%; p = 0.807), indicating that the early inflammatory response to submucosal laser energy is not negligible even though no tissue is excised. These findings accord with those of Zhang et al. [21], whose network meta-analysis showed that tissue-preserving procedures are generally associated with lower rates of urinary retention, fecal incontinence, and anal stenosis than conventional Milligan-Morgan hemorrhoidectomy. Kavraal et al. [22] similarly reported an overall complication rate of 6.1% after hemorrhoidal laser ablation using a 1470 nm diode laser in 98 patients, with no anal stenosis or fecal incontinence.

Continence

Minor continence disturbance at three months was infrequent, was limited to occasional flatus incontinence, and did not differ meaningfully between the three techniques, occurring in two laser patients (3.3%), two open patients (3.3%), and no stapled patients (p = 0.548). No patient in any group experienced incontinence to liquid or solid stool, and no difference was demonstrable after adjustment. It is worth noting that the stapled group, in which the staple line lies closest to the sphincter complex and in which continence concerns have historically been raised, recorded no continence disturbance at all. Wexner scores were obtained preoperatively and at three months in all 180 patients, including those in the stapled group, since stapled hemorrhoidopexy is capable of affecting continence and should not be reported as inapplicable. With only four events across the whole study, however, these data cannot discriminate between techniques and are best read as evidence that none of the three procedures caused clinically significant sphincter dysfunction within the observation period. This is consistent with published series reporting that clinically significant fecal incontinence is rare after all three operations when the sphincter complex is not divided [21,22].

Recurrence

At three months, recurrence occurred in two laser patients (3.3%) and two stapled patients (3.3%) and in none of the open group, a difference that was not statistically significant (p = 0.548). All four recurrences arose in the two non-excisional arms, and all four patients rated their outcome as fair or poor. Excisional surgery removes diseased tissue completely and is expected, on mechanistic grounds and on existing long-term evidence, to provide durable control, while laser and stapled techniques, which coagulate or reposition tissue rather than excise it widely, may carry a higher recurrence risk in bulky advanced disease [22,23]. Cemil et al. [23], comparing LHP and Milligan-Morgan hemorrhoidectomy for Grade 2 and 3 disease, found no significant difference in recurrence at six months, at 4.2% vs. 2.3%, consistent with the nonsignificant difference observed here.

The present data cannot, however, support any claim about long-term recurrence or radical cure. Three months is too short an interval to evaluate recurrence after hemorrhoid surgery, which characteristically presents after 12 months or later, and the absolute number of events was very small. The zero recurrence rate in the open group is therefore a short-term observation rather than evidence of superior long-term disease control. Surveillance beyond 12 months is required to determine whether any recurrence advantage exists or is sustained [24].

Patient satisfaction

Satisfaction at three months was highest in the laser group (n = 53, 88.3% good or excellent), intermediate in the stapled group (n = 48, 80.0%), and lowest in the open group (n = 36, 60.0%), with an overall p < 0.001. The strength of the evidence differs across the pairwise comparisons and should not be overstated. Only the laser-vs.-open difference survived Bonferroni adjustment (+28.3%, 95% CI +12.8 to +42.3; p = 0.002); the stapled-vs.-open difference did not (+20.0%, 95% CI +3.6 to +35.0; adjusted p = 0.083), and laser and stapled hemorrhoidopexy were not significantly different from one another (+8.3%, 95% CI -5.0 to +21.5; adjusted p = 0.952). The adjusted odds of good or excellent satisfaction after laser treatment were high (adjusted OR 9.18, 95% CI 2.70 to 47.77), but the width of that interval reflects the very small number of dissatisfied patients in the laser arm, and the point estimate should be read with that imprecision in mind. When the full four-category ordinal scale was modeled rather than dichotomized, the advantage for LHP was confirmed at a more modest magnitude (adjusted OR 5.54, 95% CI 2.59 to 11.85) and a satisfaction benefit for stapled hemorrhoidopexy emerged that the dichotomized analysis had not detected (2.48, 95% CI 1.16 to 5.29). The most defensible reading of these data is therefore that both minimally invasive techniques were rated more favorably than open hemorrhoidectomy and that the present sample does not reliably distinguish between them.

Satisfaction is a composite patient-centered outcome integrating pain, speed of recovery, resumption of daily activity, and freedom from complications, and its assessment at both POD7 and three months separates early comfort from later outcome. The same ordering was already apparent at POD7, when good or excellent ratings were given by 86.7% of laser, 76.7% of stapled, and 51.7% of open patients. The four patients who recurred all rated their outcome as fair or poor and are counted within the fair or poor category of their groups, so the group satisfaction figures already account for treatment failure; high overall satisfaction in the laser and stapled arms alongside a small number of recurrences in those arms is therefore not contradictory. Dursun et al. [17] reported 91.1% satisfaction at six weeks after LHP, consistent with the present findings, and Loutfy et al. [25], comparing LHP with harmonic scalpel hemorrhoidectomy for Grade III-IV disease, reported significantly higher satisfaction in the laser group at six weeks. The lower satisfaction in the open group most likely reflects the greater pain burden and the extended recovery, a pattern consistently observed in the comparative literature [18,19].

Cost considerations

Cost is a material consideration in this setting, is also a source of selection bias, and deserves explicit statement. Open hemorrhoidectomy requires no specialized consumables. Both minimally invasive techniques carry a substantial additional device cost, but that cost falls on different parties. At this center, the diode generator is institutional capital equipment, and the single-use radial fiber is also supplied by the institution, so LHP imposed no out-of-pocket device cost on the patient. The circular stapler, by contrast, is a single-use device purchased by the patient, and it appreciably increases the cost of stapled hemorrhoidopexy to the individual. The burden of LHP is therefore carried by the institution and that of stapled hemorrhoidopexy by the patient, and this asymmetry is the likely mechanism by which affordability became a determinant of allocation in this study. Three of the outcomes measured here bear directly on the economic balance. Compared with open hemorrhoidectomy, LHP occupied 17.20 fewer minutes of theater time, shortened hospital stay by 1.50 days, and returned patients to usual activity 9.68 days earlier; the corresponding savings for stapled hemorrhoidopexy were 10.50 minutes, 0.90 days, and 6.55 days. LHP also occupied 6.70 fewer theater minutes and 0.60 fewer bed-days than stapled hemorrhoidopexy. Current guidance acknowledges that procedure choice must weigh device cost against these benefits [9,12,13]. Nonetheless, affordability influenced which procedure some patients received, so the treatment groups may differ in socioeconomic characteristics in ways that the measured variables capture only partially. A formal cost-effectiveness analysis, incorporating unit costs for theater time, bed occupancy, consumables, and lost earnings, was not undertaken and is a reasonable objective for future work.

Clinical implications

The study supports individualized surgical decision-making rather than uniform adoption of any single technique. LHP was associated with the most favorable early postoperative profile and may reasonably be preferred when early comfort, rapid return to work, minimal operative time, short hospitalization, and short-term satisfaction are the treatment priorities, provided the disease is suitable for a non-excisional technique, and the cost is acceptable to the patient. Its measured advantage over stapled hemorrhoidopexy, however, was confined to operative duration, hospital stay, pain on the first postoperative day, and time to resumption of activity; the two techniques did not differ significantly in satisfaction, complications, continence, or recurrence at three months. Stapled hemorrhoidopexy is therefore best regarded as a sound minimally invasive alternative rather than a second-best option and is particularly suited to circumferential mucosal prolapse. Open hemorrhoidectomy retains a central role, particularly in bulky Grade IV disease with a significant external component and in acute or complicated presentations for which non-excisional treatment is unsuitable; this role rests on established long-term evidence and guideline recommendations [9,11] rather than on the three-month data presented here, which cannot address durability. These considerations are consistent with the best clinical practice recommendations of the LHP Recommendation Development Group [13] and the American Society of Colon and Rectal Surgeons clinical practice guidelines [9].

Limitations

Allocation was not randomized but depended on clinical suitability, device availability, affordability, and patient preference, so selection bias and confounding by indication are likely; adjusted and propensity-score analyses reduce but cannot eliminate this bias, and residual confounding by hemorrhoid morphology, external component, and ability to pay may persist. Follow-up was limited to three months, which is inadequate for recurrence, late stenosis, or long-term function, so no conclusion about durability or radical cure can be drawn. The study was conducted at a single tertiary center in North India, and 220 of 400 screened patients were ineligible for elective enrollment, limiting external validity; emergency presentations were excluded, so the findings do not apply to acute hemorrhoidal surgery.

Complete blinding was not feasible, so patient-reported outcomes may be subject to expectation bias. Satisfaction was rated on an unvalidated four-category scale, no disease-specific quality-of-life instrument was used, and recovery time was self-reported and influenced by occupation and by financial pressure to return to work. The study was powered only for VAS at POD1; event numbers for stenosis, recurrence, bleeding, urinary retention, and continence disturbance were small, so type II error cannot be excluded, and the absence of a demonstrated difference in these outcomes should not be read as evidence of equivalence. For the binary outcomes, the unadjusted CIs and the Bonferroni-adjusted p-values do not always agree, and the discrepancy between the unadjusted and adjusted analyses of urinary retention illustrates the fragility of estimates based on few events; these secondary findings are hypothesis-generating rather than confirmatory. Findings relate to one 1470 nm diode platform and one circular stapler and may not extrapolate to other devices. Finally, no formal cost-effectiveness evaluation was performed.

## Conclusions

In this prospective observational study of 180 patients with Grade III and Grade IV hemorrhoids, all three surgical modalities, namely open hemorrhoidectomy, LHP, and stapled hemorrhoidopexy, proved safe and effective in the short term, with no complication exceeding Clavien-Dindo grade II, no reoperation, and no mortality in any arm. The three techniques did not differ significantly in postoperative bleeding, local edema, anal stenosis, continence, or three-month recurrence. They differed substantially, however, in the early postoperative course, and those differences were large enough to be clinically meaningful rather than merely statistical. LHP delivered the most favorable early recovery profile. It achieved the shortest operative duration (22.4 ± 4.5 minutes), the briefest hospital stay (1.2 ± 0.4 days), the lowest pain scores on the first postoperative day (adjusted mean difference -3.48 vs. open hemorrhoidectomy, 95% CI -3.97 to -2.99; a 46% relative reduction), the earliest return to usual daily and occupational activity (9.66 days sooner, 95% CI 8.21 to 11.11), the lowest adjusted odds of urinary retention (0.22, 95% CI 0.04 to 0.86), and the highest proportion of patients rating their outcome as good or excellent at three months (88.3% vs. 60.0%; risk difference +28.3%, 95% CI +12.8 to +42.3). The operative time, hospital stay, pain, and recovery advantages were unchanged after multivariable adjustment and after propensity-score-weighted sensitivity analysis. The urinary retention result, by contrast, rests on 19 events in total and was not significant before adjustment and should be regarded as provisional. Stapled hemorrhoidopexy occupied a consistent intermediate position, offering substantial gains in operative efficiency, early comfort, and recovery over open surgery (operative time 10.50 minutes faster; hospital stay 0.90 days shorter; VAS at POD1, adjusted mean difference -2.37; recovery 6.58 days sooner), and by the end of the first postoperative week, its pain scores were statistically indistinguishable from those following laser treatment. Its satisfaction, complication, continence, and recurrence outcomes did not differ significantly from those of LHP, so the two minimally invasive techniques are best regarded as comparable options separated chiefly by operative duration, hospital stay, pain on day 1, and time to return to activity. Open hemorrhoidectomy was associated with the longest operative time, longest hospitalization, and greatest early morbidity. It produced no recurrence within the observation period, but three months is far too short an interval to assess durability, and this observation must not be read as evidence of superior long-term disease control.

These findings support a graded, individualized approach to Grade III and IV hemorrhoidal disease rather than uniform adoption of any single technique. LHP is an attractive first choice for predominantly vascular disease when early comfort, rapid return to work, and patient-reported satisfaction are the treatment priorities and the cost of the device is acceptable. Stapled hemorrhoidopexy is an equally defensible minimally invasive alternative, particularly for circumferential mucosal prolapse. Open hemorrhoidectomy retains its established role in bulky Grade IV disease with a significant external component and in presentations unsuitable for a non-excisional approach. Randomized trials with follow-up beyond 12 months are the natural next step and would establish whether the marked short-term advantages demonstrated here are matched by comparable long-term durability.
